# Detection of metastases using circulating tumour DNA in uveal melanoma

**DOI:** 10.1007/s00432-023-05271-3

**Published:** 2023-08-22

**Authors:** Aaron B. Beasley, Daniël P. de Bruyn, Leslie Calapre, Zeyad Al-Ogaili, Timothy W. Isaacs, Jacqueline Bentel, Anna L. Reid, Roy S. Dwarkasing, Michelle R. Pereira, Muhammad A. Khattak, Tarek M. Meniawy, Michael Millward, Erwin Brosens, Annelies de Klein, Fred K. Chen, Emine Kiliҫ, Elin S. Gray

**Affiliations:** 1 School of Medical and Health Sciences, Edith Cowan University, Joondalup, WA Australia; 2 Centre for Precision Health, Edith Cowan University, Joondalup, WA Australia; 3 Department of Ophthalmology, Erasmus MC, 3000 CA Rotterdam, The Netherlands; 4 Department of Clinical Genetics, Erasmus MC, 3000 CA Rotterdam, The Netherlands; 5 Erasmus MC Cancer Institute, 3000 CA Rotterdam, The Netherlands; 6 Department of Molecular Imaging and Therapy Service, Fiona Stanley Hospital, Murdoch, WA 6150 Australia; 7 Perth Retina, Subiaco, WA Australia; 8 Centre for Ophthalmology and Visual Science (Incorporating Lions Eye Institute), The University of Western Australia, Crawley, WA Australia; 9 Department of Ophthalmology, Royal Perth Hospital, Perth, WA Australia; 10 Anatomical Pathology, PathWest Laboratory Medicine, Fiona Stanley Hospital, Murdoch, WA Australia; 11 Department of Radiology, Erasmus MC, 3000 CA Rotterdam, The Netherlands; 12 Department of Medical Oncology, Fiona Stanley Hospital, Murdoch, WA Australia; 13 School of Medicine, The University of Western Australia, Crawley, WA Australia; 14 Department of Medical Oncology, Sir Charles Gairdner Hospital, Nedlands, WA Australia; 15 Ophthalmology, Department of Surgery, University of Melbourne, East Melbourne, VIC Australia

**Keywords:** Uveal melanoma, ctDNA, Circulating tumour DNA, Metastasis

## Abstract

**Background:**

Approximately 50% of uveal melanoma (UM) patients will develop metastatic disease depending on the genetic features of the primary tumour. Patients need 3–12 monthly scans, depending on their prognosis, which is costly and often non-specific. Circulating tumour DNA (ctDNA) quantification could serve as a test to detect and monitor patients for early signs of metastasis and therapeutic response.

**Methods:**

We assessed ctDNA as a biomarker in three distinct UM cohorts using droplet-digital PCR: (A) a retrospective analysis of primary UM patients to predict metastases; (B) a prospective analysis of UM patients after resolution of their primary tumour for early detection of metastases; and (C) monitoring treatment response in metastatic UM patients.

**Results:**

Cohort A: ctDNA levels were not associated with the development of metastases. Cohort B: ctDNA was detected in 17/25 (68%) with radiological diagnosis of metastases. ctDNA was the strongest predictor of overall survival in a multivariate analysis (HR = 15.8, 95% CI 1.7–151.2, *p* = 0.017). Cohort C: ctDNA monitoring of patients undergoing immunotherapy revealed a reduction in the levels of ctDNA in patients with combination immunotherapy.

**Conclusions:**

Our proof-of-concept study shows the biomarker feasibility potential of ctDNA monitoring in for the clinical management of uveal melanoma patients.

**Supplementary Information:**

The online version contains supplementary material available at 10.1007/s00432-023-05271-3.

## Introduction

Uveal melanoma (UM) is a rare intraocular cancer with an incidence of 7.6 and 2–8 cases per million per year in Australia (Beasley et al. 2021) and Europe (Virgili et al. 2007), respectively. At the time of diagnosis, less than 4% of patients have detectable metastatic disease (Finger et al. 2005). Unfortunately, approximately 50% of patients (Kujala et al. 2003) will develop metastases after a median time of 3.1 years following diagnosis of the primary lesion (Chew et al. 2015). Historically, after the detection of metastases, 92% of patients will die within 2 years (Diener-West et al. 2005), with survival remaining stagnant over time (Beasley et al. 2021). Currently, surgery produces the most promising improvement in overall survival following diagnosis of metastases (Mariani et al. 2009); however, this is not commonly performed, and somewhat encouraging results have been shown when combining immunotherapies ipilimumab and nivolumab or the newly approved tebentafusp for systemic treatment of metastatic lesions (Najjar et al. 2020; Nathan et al. 2021).

The current standard of care indicates contrast-enhanced magnetic resonance imaging (MRI) or ultrasound of the liver for metastatic screening every 3–12 months, depending on molecular prognosis (NCCN 2020). Additional imaging modalities used include computerised tomography (CT) with contrast to the chest/abdomen/pelvis and positron emission tomography (PET) (Bruyn et al. 2022). These scans are time-consuming, sometimes non-specific, costly, and access to facilities may be limited for some patients. Therefore, a complementary method for detecting metastatic UM using a minimally invasive methods would be beneficial, supplementing traditional care with a multi-modal approach, with the goal of earlier detection of metastases to increase the likelihood of timely treatment or surgical interventions. Furthermore, a minimally invasive method for monitoring patient response to therapy would also offer similar benefits to current post-treatment screening, allowing for closer follow-up in between radiological scan. In this regard, circulating tumour DNA (ctDNA) appears to be a promising candidate.

ctDNA are small fragments of DNA shed into the blood as a result of apoptosis or necrosis of tumour cells (Rostami et al. 2020). Previous studies in other cancers have shown that mutations identified in ctDNA strongly reflect those of the primary tumour (Calapre et al. 2019; Kidess et al. 2015) and can be used to detect disease recurrence (McEvoy et al. 2019; Gray et al. 2015). In UM, previous research has shown that ctDNA is readily detectable in patients with metastases (Beasley et al. 2018; Bidard et al. 2014), but not in primary disease (Beasley et al. 2018). One unique feature of UM is that roughly 99% of all tumours harbour distinct recurrent, hot spots, and evolutionarily truncal mutations to *GNAQ*, *GNA11*, *PLCβ4*, or *CYSLTR2* (Johansson et al. 2020), which enables simple detection through robust, targeted assays such as droplet-digital PCR (ddPCR) if the tissue has been biopsied and tested.

Herein, we report on the assessment of ctDNA as a biomarker of metastatic disease in three UM cohorts. In Cohort A, we evaluated ctDNA detection at the time of diagnosis of primary disease as a predictor of metastatic disease. Cohort B was a prospective study monitoring patients after curative treatment of the primary UM to evaluate the suitability of ctDNA as a marker of metastatic disease. Cohort C was used to assess changes in ctDNA levels in patients with metastatic UM treated with immunotherapies.

## Materials and methods

### Patients and samples

A retrospective cohort of 30 patients (Cohort A) previously described (Beasley et al. 2018) was analysed to determine the association between ctDNA levels at diagnosis of the primary tumour and survival. Patients were enrolled into the study from March 2014 and November 2016, from the Lions Eye Institute and Royal Perth Hospital in Western Australia.

For prospective analysis, 179 patients (Cohort B) with primary UM diagnosed by clinical and ultrasound examination performed by a specialist ophthalmologist were enrolled and monitored for the development of metastases. Forty-eight patients were recruited in Western Australia from Perth Retina, Lions Eye Institute, and Royal Perth Hospital, between April 2014 and March 2022. Another 131 patients were recruited from Erasmus MC and the Rotterdam Eye Hospital, Rotterdam, The Netherlands, between August 2017 and February 2021.

Six patients with metastatic UM (Cohort C) with known tumour mutations were recruited from oncology services at Sir Charles Gairdner Hospital and Fiona Stanley Hospital in Perth, Western Australia, between October 2014 and March 2019.

Written and informed consent was obtained from all patients and healthy participants under approved Human Research Ethics Committee protocols from Edith Cowan University (No. 11543 and No. 18957) and Sir Charles Gardner Hospital (No. 2013-246 and No. RGS0000003289), Western Australia; and Erasmus MC and Rotterdam Eye hospital (MEC-2009-375), The Netherlands.

### Blood collection and processing

#### Perth

Blood was collected in K2-EDTA (BD Biosciences, Franklin Lakes, NJ) or Cell-Free DNA BCT (Streck, La Vista, NE) tubes. Blood isolated from EDTA tubes was stored at 4 °C and processed within 24 h, and blood from Streck tubes was processed within 48 h.

Plasma was isolated by centrifugation at 300*g* at 4 °C for 20 min, followed by centrifugation at 4500*g* at 4 °C for 10 min for the removal of platelets. Plasma was then stored at − 80 °C until extraction.

#### Rotterdam

Blood was collected in K2-EDTA (BD Vacutainer systems, Plymouth, UK) or Cell-Free DNA BCT (Streck) tubes. Blood isolated from EDTA tubes and Streck tubes was processed within 24 h.

Plasma derived from K2-EDTA BCT was isolated by centrifugation at 3500*g* for 10 min at 4 °C, followed by centrifugation at 17,000*g* for 10 min at 4 °C. Plasma derived from Streck BCT was isolated by centrifugation at 300*g* for 20 min, followed by 19,800 g for 10 min at 4 °C. Plasma was then stored at − 80 °C until extraction.

### Genetic analysis of tumours

#### Perth

Tumour mutations were identified by targeted NGS or using droplet-digital PCR assays (Bio-Rad, Hercules, CA) for mutations common in UM, *GNA11/GNAQ* Q209L/P, *GNA11* R183C*, GNAQ* R183Q, *PLCβ4* D630Y/F*, CYSLTR2* L129Q, and *MAP2K1 P124S*, as previously described (Calapre et al. 2019).

#### Rotterdam

Targeted NGS was performed on fresh and formalin-fixed paraffin-embedded tumour tissue (FFPE). For fresh tissue, DNA was isolated using the QIAamp DNA mini kits (Qiagen, Hilden, Germany) according to the manufacturer’s specifications. For FFPE tissues, DNA was isolated using lysis buffer (Promega, Madison, WI) and 5% Chelex (Bio-Rad), as reported previously (Smit et al. 2018). NGS was performed using the IonTorrent (Thermo Fisher Scientific, Waltham, MA) platform with a custom panel consisting of, among others, *GNAQ*, *GNA11*, *EIF1AX*, *SF3B1,* and *BAP1*, as reported previously (Smit et al. 2018).

### Cell-free DNA extraction and circulating tumour DNA testing

Cell-free DNA (cfDNA) was extracted from plasma using a QIAamp Circulating Nucleic Acid kit (Qiagen) according to the manufacturer’s specifications and stored at − 80 °C until use. Extraction volumes ranged from 4–5 mL for Perth and 2–4 mL for Rotterdam cohorts. Elution volume was standardised to 40 µL. Circulating tumour DNA was quantified using the ddPCR and PrimePCR assays (Bio-Rad) for the following genes and mutations: *GNA11* Q209L*, GNAQ* Q209L/P*, GNAQ* Q209P-R210 = , *GNA11* R183C, *PLCB4* D630Y/F, and *MAP2K1 P124S* (Supplementary Tables 1 and 2). Droplets were generated using an Automated Droplet Generator (Bio-Rad), amplified using a C1000 Touch Thermal Cycler (Bio-Rad), and analysed using a QX200 system (Bio-Rad). Each gene assay on each run used a mutation-positive control, a healthy (wild-type) control, and a no-template control. QuantaSoft Analysis Pro (V 1.0.596.0525, Bio-Rad) was used for data analysis as reported previously (Beasley et al. 2018). Samples from the plasma of healthy participants were used to determine the specificity of each assay.

### Statistical analysis

Overall survival (OS) was defined as survival time from diagnosis of the primary lesion or metastatic lesion, where appropriate. Progression-free survival (PFS) was defined as survival time from diagnosis of the primary lesion to detection of metastases.

ctDNA cut-off for the cohorts was based on positivity. Differences in survival were tested using Cox-regression (*coxph*) using the survival package (v3.4-0) in R, and survival was plotted using the survminer (v0.4.9) package. *χ*^2^ was used to test the presence of ctDNA and metastases using the *chisq.test* from the stats package (v4.2.2) in R. Fisher’s exact (*fisher.test*) or Wilcoxon rank sum tests (*wilcox.test*) were used to compare the differences between ctDNA cohort characteristics in R using the stats package. ctDNA and tumour volume were log transformed. Normality was checked using Shapiro–Wilk (*shapiro.test*) in R using the stats package followed by calculation of Pearson correlation coefficient (*r*) to correlate tumour volume and ctDNA levels using the rstatix (*cor_test*, v0.7.2) and ggplot2 (v3.4.1) packages, stylised with ggprism (v1.0.4). Results with *p* < 0.05 were considered statistically significant. Base R stats package was version 4.2.2. ctDNA monitoring graphs were generated in GraphPad Prism (v9.4.1, GraphPad, San Diego, CA), and significant changes in ctDNA levels were determined using Poisson statistics with *poisson.test* from the stats package in R.

## Results

### Circulating tumour DNA levels in primary uveal melanoma are not associated with survival

We analysed the survival of a cohort of 30 patients (Cohort A) from our previous study (Beasley et al. 2018), to evaluate whether ctDNA detection prior to treatment of the primary disease was predictive of shorter OS or PFS. In this cohort, we used a tumour agnostic approach due to lack of biopsied tumours. We screened using a panel of common UM mutations: *GNA11* R183C/Q209L, *GNAQ* R183Q/Q209L/Q209P, *PLBC4* D630Y, and *CYSLTR2* L129Q. Detailed cohort characteristics can be found in the previous study (Beasley et al. 2018).

**Fig. 1:**
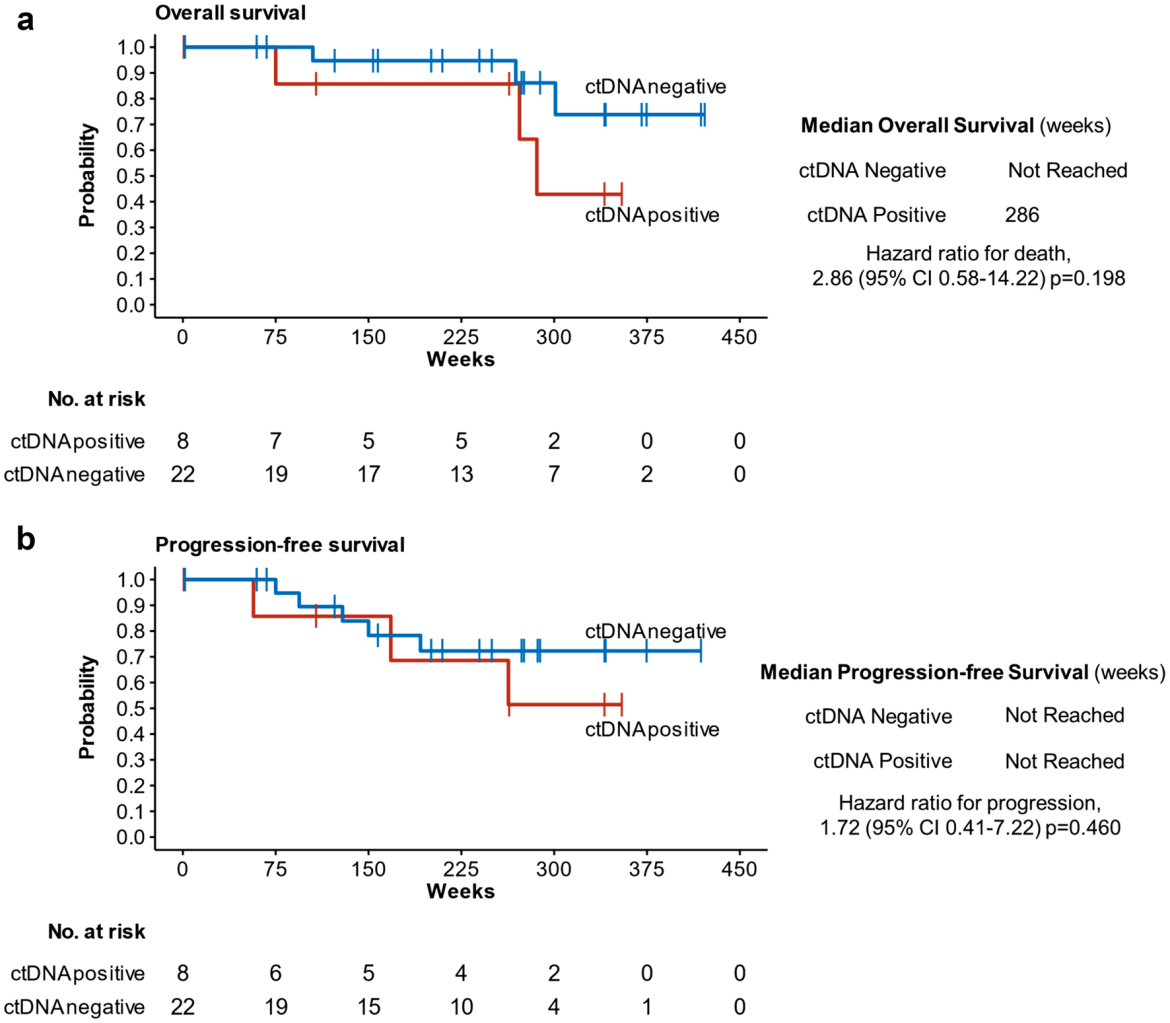
Survival in primary UM patients—Cohort A. **a** Overall and **b** progression-free survival curves based on detectability of ctDNA in primary UM patients. Hazard ratio (HR) and p value from Cox-regression

Here, we report a follow-up on this cohort with a median of 256 weeks (~ 5 years). Notably, no significant difference in OS (Fig. 1a) or PFS (Fig. 1b) was found between patients with detectable (*n* = 8, median (range) = 3.3 (1.6–29) copies/mL) and undetectable ctDNA at the time of treatment of the primary disease.

### Circulating tumour DNA is detectable at radiological progression and is associated with worse survival

**Fig. 2:**
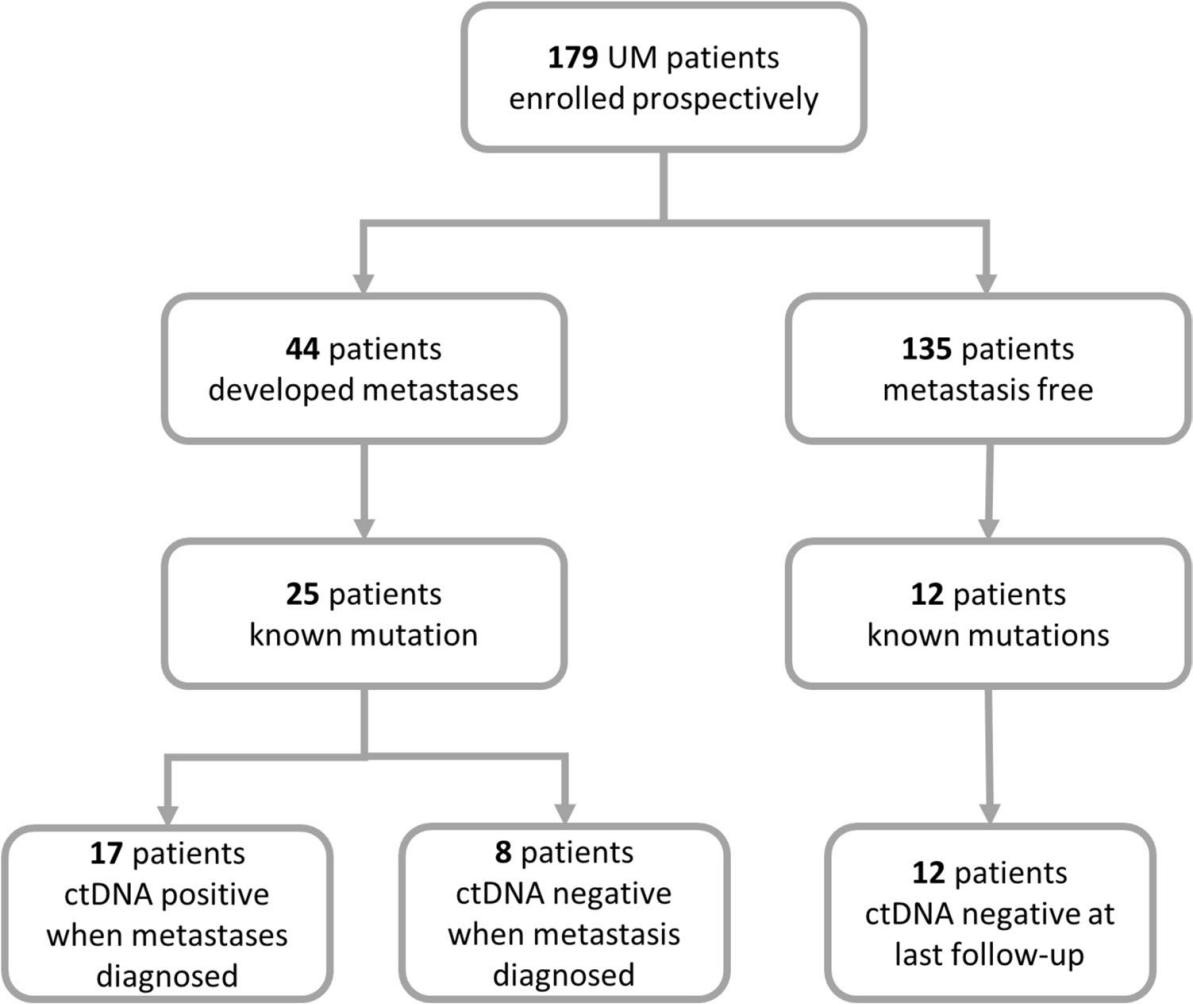
Flow chart of prospectively recruited patients in Cohort B. 179 patients were recruited to the study at the diagnosis of their primary lesion, of which 44 developed metastases during the study period, including 25 with known mutations. Of the 135 patients with no metastases, 12 were tested on their routine blood draw for ctDNA for use in the *χ*^2^ test

We monitored 179 patients (Cohort B) diagnosed with primary UM for the development of metastases (Fig. 2). Of these patients, 44 progressed with metastatic disease (median time to progression: 88 weeks). Here, we employed a tumour informed approach for ctDNA detection, thus only 25 that had a known hot spot driver mutation identified in their tumour proceeded with ctDNA analysis. Within this cohort, the median age of diagnosis was 61 (37–86); 56% were female; 80% had a choroidal anatomical location; 76% were high risk, 8% low or intermediate, and 16% were unknown; 84% had a driver mutation to either *GNAQ* or *GNA11* Q209; 52% were treated with enucleations, 36% with Iodine^125^ plaques, and 12% with stereotactic radiation; and lastly at the time of writing, 52% of patients had died to UM metastases. Similarly, of the 135 patients without progression by the time of analysis (median follow-up time: 103 weeks), 12 had known mutations derived from the analysis of their primary tumour and were tested for ctDNA in plasma at their last follow-up.

ctDNA was detectable in 17/25 (68%) of the plasma samples collected at the time of diagnosis of metastatic disease. In contrast, none of the 12 metastasis-free cases had detectable ctDNA. Thus, we found ctDNA detection to be significantly associated with the presence of metastases (*χ*^2^ = 12.48, *df* = 1, *p* < 0.001) with a sensitivity of 68%, a specificity of 100%, a positive predictive value of 100%, and a negative predictive value of 60%.

Within the 25 patients that developed metastases, 11 had regular blood collections every ~ 3 months. Of those, 5/11 (45%) had ctDNA detectable with a median lead time of 4.4 months (range 2.5–5.8) before clinical manifestation of metastatic disease.

**Fig. 3:**
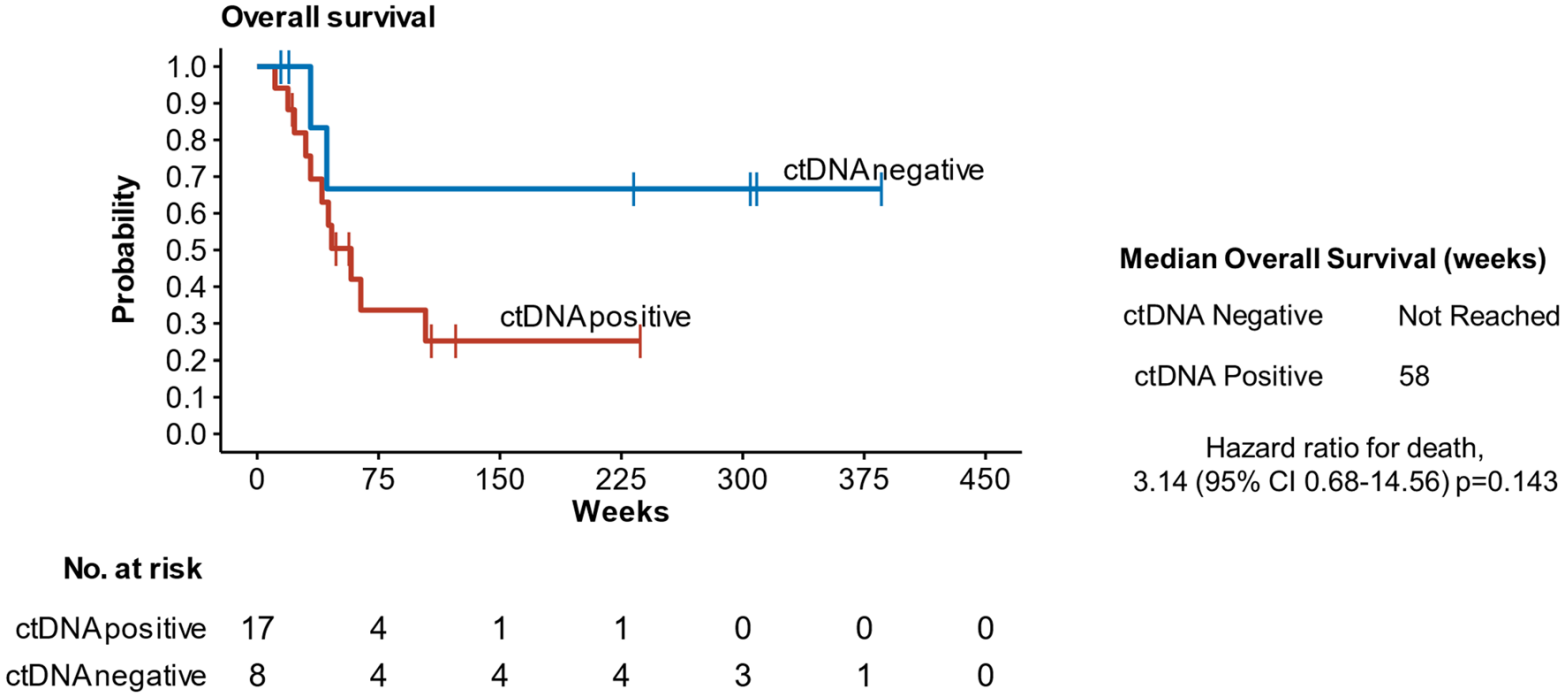
Overall survival in metastatic UM patients. Metastatic UM patients were separated into two groups based on positivity/negativity of ctDNA. Hazard ratios (HRs) and p values were calculated from Cox-regression

We found no statistically significant differences in univariate OS between patients with detectable ctDNA in plasma and those without at the time of diagnosis of metastatic disease (Fig. 3, Table 1, Supplementary Table 3) with a trend towards shorter OS in the ctDNA positive group observed (Fig. 3). Notably, in a multivariate Cox model a significantly worse OS post-diagnosis of metastases was observed for patients with positive ctDNA (HR = 15.8, 95%, CI 1.7–151.2, *p* = 0.017) and ciliary body as the primary tumour site (HR = 5.77, 95% CI 1.1–29.3, *p* = 0.034) (Table 2).

**Table 1:** Clinical characteristics of the metastases detection cohort

Variable	Unit	Group				*p* value
		ctDNA positive		ctDNA negative		
		Value	Deviation (range)	Value	Deviation (range)	
*Age*	*n*	17		8		0.07
Mean	Years	57.1	10.5 (37–71)	65.9	11.2 (50–86)	
Median	Years	60	16	64.5	16.5	
*Sex*	*n*	17		8		0.23
Male	*n*	9		2		
Female	*n*	8		6		
*Anatomical Location*	*n*	17		8		1
Choroid	*n*	13		7		
Ciliary Body	*n*	4		1		
*Eye*	*n*	17		8		1
Left	*n*	10		4		
Right	*n*	7		4		
*Apical Height (primary)*	*n*	16		8		0.53
Mean	mm	7.4	4.0 (1–14)	7.2	3.2 (2–13)	
Median	mm	8	5.8	7.3	3.2	
*Largest Basal Diameter (primary)*	*n*	16		8		0.15
Mean	mm	14.5	2.8 (10–20)	16.1	2.4 (12–20)	
Median	mm	14.5	4.1	16.4	2	
Risk	*n*	14		7		0.1
*Low/Intermediate*	*n*	0		2		
High	*n*	14		5		
Primary Therapy	*n*	17		8		1
Radiation	*n*	8		4		
Enucleation	*n*	9		4		
*ctDNA (metastases)*	*n*	17		8		na
Mean	copies/mL	572.4	2107.9 (1.6–9000)	0		
Median	copies/mL	19.5	75.3	0		
*Mutation*	*n*	17		8		na
MAP2K1 c.370C > T	*n*	0		1		
GNA11 c.626A > T	*n*	8		5		
GNAQ c.626A > C	*n*	6		1		
GNAQ c.626delinsCAAGA	*n*	1		0		
PLCB4 c.1888 delinsTT	*n*	2		0		
*Location of Metastases^*	*n*	17		8		0.85
Liver	*n*	16		6		
Lungs	*n*	3		2		
Other	*n*	4		1		
*Number of Metastatic Sites*	*n*	17		8		1
1	*n*	13		7		
> 1	*n*	4		1		
*MTB*	*n*	7		5		0.048
Mean	TLG	82.4	84.2 (59–267.5)	14.8	13.4 (0–36.5)	
Median	TLG	42.4	80.9	15.5	18.7	
*MTV*	*n*	7		5		0.11
Mean	mm^3^	18.7	16.2 (1.7–50.2)	4.8	3.7 (0–9.5)	
Median	mm^3^	11	21.3	5.4	6.6	
*TV*	*n*	6		2		0.43
Mean	mm^3^	164.6	312.2 (3.3–861.5)	12.7	4.3 (8.4–16.9)	
Median	mm^3^	22.9	39.9	12.7	4.3	

*TLG* total lesion glycolysis, *MTB* metabolic tumour burden, *MTV* metabolic tumour volume, *TV* tumour volume, *na* not applicable

^Note patients can have more than one location of metastases

**Table 2:** Survival analysis of metastatic patients for overall survival

Variable	Group	Value	Univariate HR (95% CI, *p* value)	Multivariate HR (95% CI, *p* value)
Age	Mean (SD)	59.9 (11.7)	1.04 (0.98–1.10, *p* = 0.179)	1.07 (0.99–1.15, *p* = 0.069)
Sex	F	14 (56)	–	–
	M	11 (44)	1.10 (0.37–3.27, *p* = 0.866)	0.46 (0.12–1.79, *p* = 0.264)
Anatomical Location	C	20 (80)	–	–
	CB	5 (20)	**4.48 (1.20–16.65, *p* = 0.025)**	**5.77 (1.14–29.28, *p* = 0.034)**
Number of metastatic sites	1	20 (80)	–	–
	> 1	5 (20)	2.24 (0.61–8.24, *p* = 0.225)	8.57 (0.81–91.07, *p* = 0.075)
ctDNA	Negative	8 (32)	–	–
	Positive	17 (68)	2.94 (0.65–13.37, *p* = 0.163)	**15.8 (1.65–151.23, *p* = 0.017)**

Bold text indicates statistical significance

*HR* hazard ratio, *CI* confidence interval, *SD* standard deviation, *F* female, *M* male, *C* choroid, *CB* ciliary body; *ctDNA* circulating tumour DNA

### Levels of circulating tumour DNA are associated with increased tumour burden

**Fig. 4:**
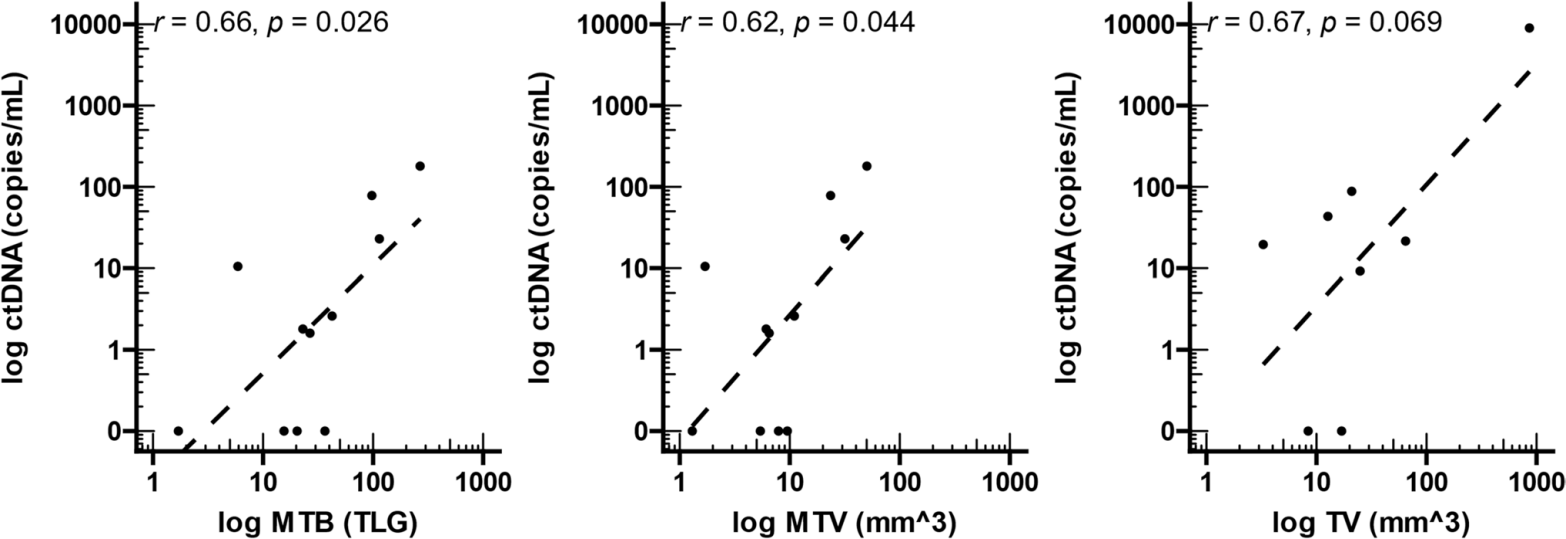
Correlation between levels of log-transformed ctDNA levels and total lesion size. **a** Metabolic tumour burden (MTB), **b** metabolic tumour volume (MTV), and **c)** tumour volume (TV) were compared to ctDNA levels. *r* and *p* values were calculated using Pearson’s correlation coefficient. TLG—total lesion glycolysis

Given the variance in ctDNA detectability between patients, we analysed the correlation of ctDNA levels with metabolic disease burden (MTB) and volume (MTV) in 11 patients for whom PET scan results were available (Perth) and tumour volume (TV) in 8 patients by MRI (Rotterdam). We found a statistically significant correlation between the levels of ctDNA and MTB (*r* = 0.66, *p* = 0.026) and MTV (*r* = 0.62, *p* = 0.044) (Fig. 4a, b). However, despite the trend, no statistically significant correlation was found between ctDNA and MRI-based tumour size (Fig. 4c).

### Circulating tumour DNA and response to immunotherapy

**Fig. 5:**
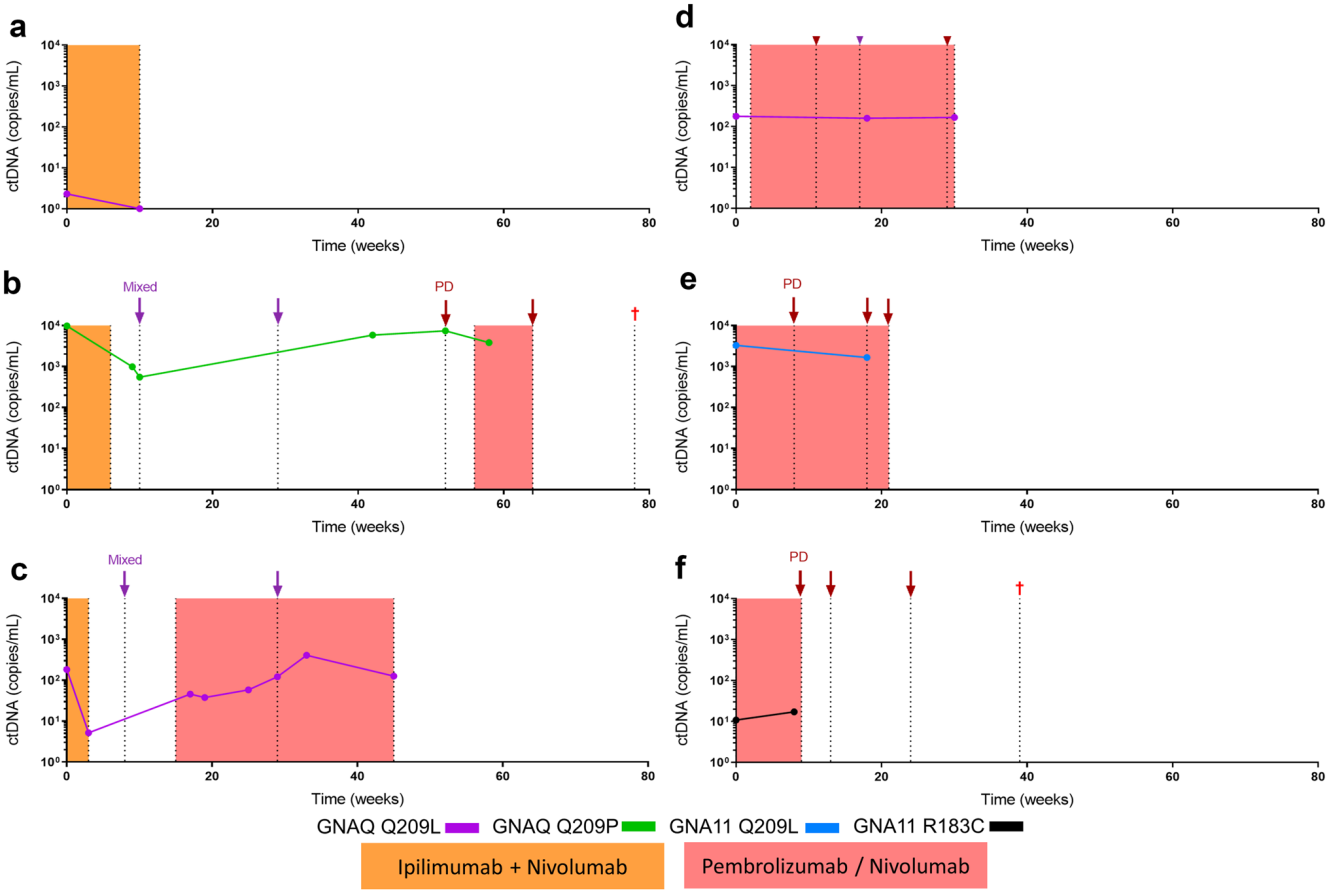
The levels of ctDNA during treatment. **a–f** The levels of ctDNA inpatients with clinically evident metastatic disease. ctDNA was assessed using tumour confirmed mutations to *GNAQ* Q209L, *GNAQ* 209P, or *GNA11* R183C as noted on each figure. The dotted black lines indicate the exact time of events in the patient’s monitoring and treatment timeline. Red arrows (PD) indicate progressive disease; purple arrows (mixed) indicate mixed response to therapy. Grey arrows indicate treatment time point, with the treatment described above. Orange box indicates combination immunotherapy. Salmon box indicates single agent immunotherapy. The red cross indicates that the patient passed-away from uveal melanoma metastases. Coloured lines indicate levels of ctDNA

Six patients with metastatic UM were recruited into the study prior to the initiation of immunotherapy (Table 2). Of these patients, three received initial combination ipilimumab plus nivolumab immunotherapy, and three received single agent pembrolizumab. A total of 22 plasma samples were tested for ctDNA throughout an average monitoring period of 62 weeks. For the three patients treated with combination immunotherapy, treatment was ceased prior to completion of all four infusions due to toxicities (Fig. 5a–c). Notably, analysis of their plasma revealed a reduction in ctDNA to undetectable or a log lower when compared to baseline levels: (a) 2.3 to 0 copies/mL, (b) 9,800 to 989 copies/mL (*p* < 0.001), and (c) 183 to 5.3 copies/mL (*p* < 0.001). In contrast, no log change in ctDNA was observed in the three patients treated with single agent pembrolizumab (Fig. 5d–f, Table 3), with only patient (Fig. 5e) having a significant reduction of ctDNA levels (3300 to 1660 copies/mL, *p* < 0.001). Radiological responses were noted on each graph.

**Table 3:** Clinical Characteristics of the Treatment Response Cohort

PID	Age	Sex	Site of Metastases at Baseline	Mutation	Metastatic risk	FU (weeks)	ctDNA reduction (*p* value)
a) PER17	62	F	Lung	GNAQ Q209L	Low	72	1
b) PER18	45	M	Liver, Lungs, Lymph Nodes, Muscle, Bone, Bone Marrow, Pleura	GNAQ Q209P	Unknown	78	< 2.2e-16
c) PER19	68	M	Liver, Lymph Nodes	GNAQ Q209L	Unknown	45	< 2.2e-16
d) PER20	73	M	Liver, Subcutaneous, Retroperitoneum	GNAQ Q209L	Unknown	144	0.4742
e) PER21	68	M	Lymph nodes, Abdomen, Pleura	GNA11 Q209L	Unknown	21	< 2.2e-16
f) PER22	82	F	Liver, Lung, Lymph nodes	GNA11 R183C	High	10	0.3636

*PID* patient identification number, *FU* follow-up

## Discussion

In this report, we describe retrospective analyses of ctDNA from diagnosis to metastases and during monitoring of metastatic disease. Tumour specific mutations were used to detect ctDNA at the time of radiological diagnosis of the metastatic tumour and to track changes in disease levels during treatment. Overall, we found that ctDNA was associated with the presence and volume of the metastases.

Retrospective analysis of data from our previous study (Beasley et al. 2018), with updated follow-up, revealed no difference in survival between patients with detectable or undetectable ctDNA. Unfortunately, this study was originally designed as a cross-sectional study and thus patient follow-up was not homogenous across the cohort. This resulted in many censored events as lost to follow-up. Similarly, a recent prospective study that found ctDNA at diagnosis did not appear to have any impact on survival, and only longitudinal increases/detection (i.e. evidence of metastases) had worse survival (Francis et al. 2022).

A recent study (Guin et al. 2021) monitored 21 UM patients that developed metastases or local recurrence using deep next-generation sequencing. Of these, 17 (81%) had detectable ctDNA at clinical manifestation of re-occurrence. While this is higher than the 68% detected in our study, the majority of patients in that study had metastases diagnosed clinically, while in this study radiological evidence of disease was detected in mostly asymptomatic patients. Furthermore, 14 patients who were ctDNA negative had unknown causes of death (Guin et al. 2021). This might lead to an overrepresentation of positive cases, masking the true sensitivity and detection rate.

By prospectively monitoring for metastases, we observed that patients with detectable ctDNA at clinical diagnosis of metastases had worse overall survival when compared to patients who did not. This reinforces previous studies in stage IV UM (Bidard et al. 2014; Ny et al. 2021; Mariani et al. 2023) where high levels of ctDNA correlated with worse outcomes. We also observe the univariate effect of increasing ctDNA levels associating with worse survival in our study. For example, stratifying patients with > 10 or ≤ 10 copies of baseline ctDNA leads to a significant survival difference. However, the clinical relevance of such a cut-off is not clear and further larger-scale studies are required for this analysis.

Notably in our study, for most patients with undetectable ctDNA the disease has continued to be effectively controlled through repeated tumour resections, radiation, and immunotherapy with continuing long-term survivals of up to 7.3 years after diagnosis of metastases. Therefore, combinations of ctDNA levels and other clinical characteristics in the metastatic setting might provide reliable prognostic information for patients.

Studies have shown that patients in whom ctDNA was undetectable at baseline or became undetectable during anti-PD1 therapy had significantly better overall survival in multiple cancers (Cabel et al. 2017; Lee et al. 2017). In UM, this has been observed in patients undergoing treatment with tebentafusp, where patients with ctDNA clearance had 100% 1-year OS versus 57% in patients with increased ctDNA (Shoushtari et al. 2021). Here, we showed that in three patients undergoing combination immunotherapy, ctDNA drastically reduced, whereas this was not the case in patients undergoing single-agent anti-PD1 treatment. Given the low sample size, comparison of ctDNA reductions between combination and single agent immunotherapy is difficult to interpret. Previous studies have shown little to no benefit in UM using of single agent ipilimumab (Heppt et al. 2017) or pembrolizumab (Rossi et al. 2019). On the other hand, recent studies showed small overall survival benefit with combination immunotherapy (Najjar et al. 2020; Piulats et al. 2021). Moreover, the observed median OS of 15 months suggested some clinical benefit was obtained from the intervention (Najjar et al. 2020). Given that ctDNA has been associated with total disease volume in ours and other studies (Bidard et al. 2014), the remarkable reduction we observed might indeed indicate that UM may be treatable using different immunotherapies.

Recent studies have highlighted novel associations with immune checkpoints proteins that could be useful such as LAG3, TIM3, or TIGIT (Karlsson et al. 2020; Durante et al. 2020; Lin et al. 2021) due to their expression levels within tumours. Future clinical trials of agents targeting these molecules may enhance the effect of the combination of anti-CTLA and anti-PD1. It would be beneficial to include ctDNA monitoring in such studies as an early indicator of clinical benefit, as has been seen in the recent phase III tebentafusp trial (Shoushtari et al. 2021; Carvajal et al. 2022).

A limitation of our study is the small number of included participants. UM is a rare disease and to overcome sample size limitations large prospective multicentre studies need to be conducted to demonstrate the clinical utility of ctDNA monitoring. While we were able to enrol 179 patients, only 25 had tumour confirmed driver mutations and developed metastases during the study period. Confirmation of driver mutations was essential to ensure the specificity of ctDNA detection. In general, biopsies of the primary lesion are not performed, or very limited material is obtained (Beasley et al. 2022). However, lack of driver mutation knowledge might be further overcome using a multi-modal approach incorporating un-targeted methylation or fragmentomic profiles instead of mutations (Wong et al. 2023). Nevertheless, even in the metastatic setting, there is not always enough material from the biopsy used to confirm tumour type, and occasionally patients are not biopsied at this stage. This limitation could be potentially overcome using a tumour agnostic method exploiting a targeted next-generation sequencing panel that cover all UM associated mutations (Smit et al. 2018).

## Supplementary Information

Below is the link to the electronic supplementary material.

Supplementary file1 (DOCX 30 KB)

## Data Availability

Data are available upon reasonable request. Underlying data for Figs. 3 and 4 (cohort B) can be found in the supplementary.
