# Supplementary material for: Detection of metastases using circulating tumour DNA in uveal melanoma
Source: J Cancer Res Clin Oncol. 2023 Aug 22;149(16):14953–63. doi: 10.1007/s00432-023-05271-3 (PMC10602949; doi:10.1007/s00432-023-05271-3)
Supplement: Supplementary file 1 — Supplementary file1 (DOCX 30 KB) [file 432_2023_5271_MOESM1_ESM.docx]

**Supplementary Information**

**Detection of metastases using circulating tumour DNA in uveal melanoma**

Aaron B Beasley, Daniël P de Bruyn, Leslie Calapre, Zeyad Al-Ogaili, Timothy W Isaacs, Jacqueline Bentel, Anna L. Reid, Roy S Dwarkasing, Michelle R Pereira, Muhammad A Khattak, Michael Millward, Erwin Brosens, Annelies de Klein, Fred K Chen, Emine Kiliҫ, Elin S Gray

**Supplementary Table 1: Droplet digital PCR assays utilised**

| Assay | BioRAD ID |
| --- | --- |
| GNAQ p.Q209L | dHsaCP2000051 |
| GNAQ p.Q209 (WT) | dHsaCP2000052 |
| GNAQ p.Q209P | dHsaCP2506794 |
| GNAQ p.Q209 (WT) | dHsaCP2506795 |
| GNA11 p.Q209L | dHsaCP2000049 |
| GNA11 p.Q209 (WT) | dHsaCP2000050 |
| GNAQ p.R183Q (w/ WT) | dHsaMDS533896396 |
| GNA11 p.R183C (w/ WT) | dHsaMDS314447910 |
| PLCB4 p.D630F (w/ WT) | dHsaMDS452929053 |
| CYSLTR2 p.L129Q (w/ WT) | dHsaMDS189202548 |
| GNAQ p.Q209P p.R210= (w/ WT) | dHsaMDS130771439 |
| MAP2K1 p.P124S (w/ WT) | dHsaMDS641696751 |

**Supplementary Table 2: Droplet digital PCR assay specificity against healthy controls**

|  | GNAQ  Q209L | GNAQ  Q209P | GNA11  Q209L | GNAQ R183Q | GNA11 R183C | PLCB4 D630F | CYSLTR2 L129Q | GNAQ Q209P R210 | MAP2K1 P124S |
| --- | --- | --- | --- | --- | --- | --- | --- | --- | --- |
| Positives | 0/37 | 0/21 | 1/49^ | 0/14# | 0/3# | 0/11 | 0/12 | NT | 2/5^ |
| Specificity | 100% | 100% | 98% | 100% | 100% | 100% | 100% | NA | 60% |

^ - low relative fluorescence units; double positive (orange) dot; # - threshold of 3.2 copies/20 µL well; NT – not tested; NA – not applicable.

**Supplementary Table 3: Metastatic cohort data**

| **ID** | **Driver mutation** | **mUM ctDNA [copies/mL]** | **OS [wk]** | **Censor** | **MTB [TLG]** | **MTV [mm^3^]** | **TV [mm^3^]** |
| --- | --- | --- | --- | --- | --- | --- | --- |
| PER1 | GNA11 c.626A>T | 1.6 | 104 | 1 | 26.7 | 6.5 | - |
| PER2 | MAP2K1 c.370C>T | 0 | 308 | 0 | 1.7 | 1.3 | - |
| PER3 | GNA11 c.626A>T | 1.8 | 30 | 1 | 23 | 6.1 | - |
| PER4 | GNA11 c.626A>T | 0 | 304 | 0 | -^ | -^ | - |
| PER5 | GNA11 c.626A>T | 78.1 | 58 | 1 | 97.4 | 23.5 | - |
| PER6 | GNA11 c.626A>T | 0 | 232 | 0 | 15.5 | 9.5 | - |
| PER7 | GNAQ c.626A>C | 22.9 | 23 | 1 | - | - | - |
| PER8 | GNAQ c.626delinsCAAGA | 10 | 44 | 1 | - | - | - |
| PER9 | GNA11 c.626A>T | 0 | 33 | 1 | - | - | - |
| PER10 | GNAQ c.626A>C | 2.6 | 107 | 0 | 42.4 | 11 | - |
| PER11 | GNA11 c.626A>T | 0 | 385 | 0 | 36.5 | 7.9 | - |
| PER12 | PLCB4 c.1888 delinsTT | 179.7 | 33 | 1 | 267.5 | 50.2 | - |
| PER13 | GNAQ c.626A>C | 0 | 19 | 0 | 20.4 | 5.4 | - |
| PER14 | GNA11 c.626A>T | 1.6 | 56 | 0 | - | - | - |
| PER15 | GNAQ c.626A>C | 237.2 | 64 | 1 | - | - | - |
| PER16 | GNA11 c.626A>T | 10.5 | 40 | 1 | 5.9 | 1.7 | - |
| RTM1 | GNA11 c.626A>T | 2.8 | 236 | 0 | - | - | - |
| RTM2 | GNA11 c.626A>T | 0 | 14 | 0 | - | - | 8.4 |
| RTM3 | GNAQ c.626A>C | 19.5 | 48 | 0 | - | - | 3.3 |
| RTM4 | GNAQ c.626A>T | 0 | 43 | 1 | - | - | 16.9 |
| RTM5 | GNA11 c.626A>T | 9000 | 19 | 1 | - | - | 861.5 |
| RTM6 | GNA11 c.626A>T | 43.3 | 11 | 1 | - | - | 12.7 |
| RTM7 | PLCB4 c.1888 delinsTT | 21.5 | 21 | 0 | - | - | 64.6 |
| RTM8 | GNAQ c.626A>C | 9.2 | 122 | 0 | - | - | 24.9 |
| RTM9 | GNAQ c.626A>T | 88 | 46 | 1 | - | - | 20.9 |

^ – visible, but too small to be quantified via PET/CT; mUM – metastatic uveal melanoma; OS – overall survival; MTB – metabolic tumour burden; TLG – total lesion glycolysis; MTV – metabolic tumour volume; TV – tumour volume.
